# Data set for the genome-wide transcriptome analysis of human epidermal melanocytes

**DOI:** 10.1016/j.dib.2014.09.002

**Published:** 2014-10-27

**Authors:** Kirk D. Haltaufderhyde, Elena Oancea

**Affiliations:** Department of Molecular, Pharmacology, Physiology & Biotechnology, Brown University, United States

## Abstract

The data in this article contains data related to the research articled entitle Genome-wide transcriptome analysis of human epidermal melanocytes. This data article contains a complete list of gene and transcript isoform expression in human epidermal melanocytes. Transcript isoforms that are differentially expressed in lightly versus darkly pigmented melanocytes are identified. We also provide data showing the gene expression profiles of cell signaling gene families (receptors, ion channels, and transcription factors) in melanocytes. The raw sequencing data used to perform this transcriptome analysis is located in the NCBI Sequence Read Archive under Accession No. SRP039354 http://dx.doi.org/10.7301/Z0MW2F2N.

**Specifications table**

**Table:** 

Subject area	Biology
More specific subject area	Human epidermal melanocytes transcriptome
Type of data	Tables (Excel spreadsheets), Text (FASTQ sequence files)
How data was acquired	High-throughput RNA sequencing using the illumina HiSeq 2000
Data format	Raw and analyzed
Experimental factors	Melanocyte mRNA libraries were prepared using the Illumina TruSeq RNA Sample Preparation Kit
Experimental features	Samples were derived from human foreskin
Data source location	Providence, RI
Data accessibility	Data is within this article and in the NCBI Sequence Read Archive under Accession No. SRP039354 10.7301/Z0MW2F2N

**Table:** **Value of the data**.

• This data provides a comprehensive view of the melanocyte transcriptome. • We identify 166 transcript isoforms that are differentially expressed in lightly vs. darkly pigmented melanocytes that could potentially play a role in regulating melanin synthesis. • This vast gene expression dataset is organized into cell signaling gene families providing a resource for identifying novel signaling genes and pathways in melanocytes. • Having access to the raw sequencing data allows researchers to perform their own computational analysis using novel algorithms.

**Data, experimental design, materials and methods**

## 1 Human epidermal melanocyte gene and transcript isoform expression data

Supplementary Table 1: A complete data set of transcript abundances, sizes, and alternative splicing for all four HEM cDNA libraries.

Supplementary Table 2: Data set of GPCR, receptor kinase, & ion channel gene expression in HEMs.

Supplementary Table 3: A complete data set of transcription factor gene expression in HEMs.

SupplementaryTable 4: Analysis of differentially expressed isoforms in lightly versus darkly pigmented HEMs.

The raw FASTQ sequencing data used to perform this transcriptome analysis is located in the NCBI Sequence Read Archive under Accession No. SRP039354 http://dx.doi.org/10.7301/Z0MW2F2N.

## 2 Sample collection, library preparation and sequencing

Lightly and darkly pigmented primary human epidermal melanocytes (HEMs) from neonatal foreskin (Life Technologies/Gibco) were cultured in Medium 254 and Human Melanocyte Growth Supplement (HMGS2, Life Technologies/Gibco). The four HEM lines used for this study (HEM-D1, -D2, -L1, -L2) were each derived from a different donor; all lines were propagated in culture under identical conditions for ≤15 population doublings. Total HEM RNA was isolated using the mirVana miRNA Isolation Kit (Ambion) and its quality was assessed using an Agilent 2100 Bioanalyzer: RNA Integrity Number (RIN)≥8.7 for all samples, in agreement with Illumina recommended RIN≥8. cDNA libraries were prepared with 4 µg total RNA using standard Illumina protocols (TruSeq RNA Sample Preparation Kit) and resulted in cDNA fragments with 229 bp average size. Each cDNA library was sequenced with 50 bp single-read chemistry using the IIlumina HiSeq 2000 system.

## 3 Data analysis and annotation

The computational pipeline used for data analysis is shown in Supplemental Figure S1 in [1]. The quality of the raw sequencing data was checked using FastQC (http://www.bioinformatics.babraham.ac.uk/projects/fastqc/) and the quality scores of the reads for each library and for each position in the read were above 30, which is defined as high quality (see Supplemental Figure S2 in [1]). The sequencing data in FASTQ format was then mapped against NCBI build 37.2 of the human genome using Bowtie 2 (version 2.1.0.0) [2]. RNA sequencing metrics were obtained using Picard tools (http://picard.sourceforge.net/) (see Table 1 in [1]). Spliced junctions were identified using Tophat (version 2.0.8) [3] and transcript abundance estimates were performed using Cufflinks (version 2.1.1) [4]. EdgeR was used to perform differential gene expression analysis between samples with different pigmentation levels [5,6] (see Table 5 in [1]). Genes differentially expressed were considered significant if they had a FDR adjusted *p*-value<0.05 [7]. For the isoform analysis we imported the Cufflinks isoform expression data of lightly and darkly pigmented HEMs into Microsoft Excel. Using Excel functions we identified the isoforms only present in HEM-L or HEM-D and excluded those with high degree of variability (standard error mean>35% the average FPKM).
